# Engineering of an endogenous hexose transporter into a specific D-xylose transporter facilitates glucose-xylose co-consumption in *Saccharomyces cerevisiae*

**DOI:** 10.1186/s13068-014-0168-9

**Published:** 2014-11-29

**Authors:** Jeroen G Nijland, Hyun Yong Shin, René M de Jong, Paul P de Waal, Paul Klaassen, Arnold JM Driessen

**Affiliations:** Molecular Microbiology, Groningen Biomolecular Sciences and Biotechnology, University of Groningen, Zernike Institute for Advanced Materials and Kluyver Centre for Genomics of Industrial Fermentation, Groningen, The Netherlands; DSM Biotechnology Center, Alexander Fleminglaan 1, 2613 AX Delft, The Netherlands

**Keywords:** Sugar transporter, Xylose transporter, Evolutionary engineering, Bioethanol, Yeast

## Abstract

**Background:**

Engineering of *Saccharomyces cerevisiae* for the simultaneous utilization of hexose and pentose sugars is vital for cost-efficient cellulosic bioethanol production. This yeast lacks specific pentose transporters and depends on endogenous hexose transporters for low affinity pentose uptake. Consequently, engineered xylose-fermenting yeast strains first utilize D-glucose before D-xylose can be transported and metabolized.

**Results:**

We have used an evolutionary engineering approach that depends on a quadruple hexokinase deletion xylose-fermenting *S. cerevisiae* strain to select for growth on D-xylose in the presence of high D-glucose concentrations. This resulted in D-glucose-tolerant growth of the yeast of D-xylose. This could be attributed to mutations at N367 in the endogenous chimeric Hxt36 transporter, causing a defect in D-glucose transport while still allowing specific uptake of D-xylose. The Hxt36-N367A variant transports D-xylose with a high rate and improved affinity, enabling the efficient co-consumption of D-glucose and D-xylose.

**Conclusions:**

Engineering of yeast endogenous hexose transporters provides an effective strategy to construct glucose-insensitive xylose transporters that are well integrated in the carbon metabolism regulatory network, and that can be used for efficient lignocellulosic bioethanol production.

**Electronic supplementary material:**

The online version of this article (doi:10.1186/s13068-014-0168-9) contains supplementary material, which is available to authorized users.

## Background

**Figure 1:**
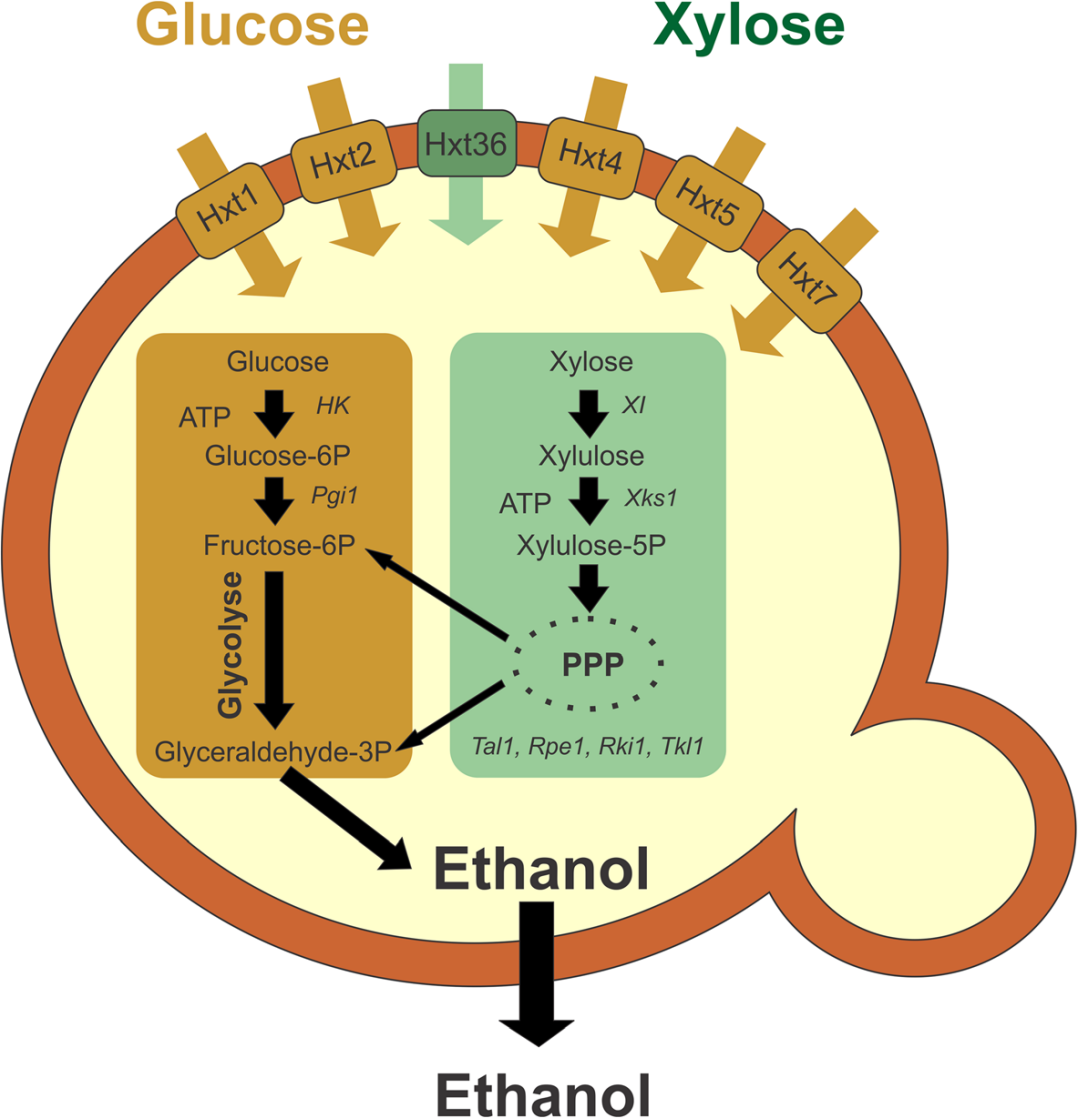
**Scheme of yeast** ***S. cerevisiae*** **able to ferment D-glucose and D-xylose** ***.*** D-glucose and D-xylose are transported into the cell via the expressed hexose transporters Hxt1-7. The expressed Hxt36 chimera in the DS68616 lineage is a result of a deletion of a C-terminal part of the *HXT3* coding sequence, the *HXT3* terminator, the *HXT6* promoter, and an N-terminal part of the *HXT6* coding sequence. The metabolism of glucose is mediated via the enzymes hexokinase (HK) and glucose 6P isomerase (Pgi1) to yield fructose-6P, which is further converted in the glycolytic pathway into ethanol. The conversion of xylose into xylulose is possible in *S. cerevisiae* via the introduction of xylose isomerase (XI), followed by the phosphorylation of xylulose into xylulose-5P by xylulose kinase (Xks1). Xylulose-5P enters the pentose phosphate pathway to eventually yield fructose-6P and glyceraldehyde-3P which can be converted into ethanol. The key enzymes of the pentose phosphate pathway (Tal1, Rpe1, Rki1, and Tki1) were overexpressed for an efficient conversion. Since the endogenous Hxt transporters show a higher affinity for D-glucose compared to D-xylose, in mixed sugar fermentation, the D-glucose is used first before the D-xylose can be metabolized. In the transporter engineered D-xylose-fermenting yeast, the specificity of the Hxt36 transporter is changed by mutagenesis, allowing preferred uptake of D-xylose relative to D-glucose.

For the last three decades biofuels produced from renewable feedstocks have received much publicity because of their potential to replace conventional fossil fuels. However, the use of readily fermentable agricultural feedstocks like sugar cane and corn for the production of bioethanol is less desirable because of its negative connotation in the “food versus fuel” debate [1]. Instead, lignocellulosic biomass from hardwood, softwood and agricultural residues is generally considered as a more sustainable source of feedstocks for biofuels [2]. A major issue in the conversion of saccharified cellulosic biomass into biofuel is the utilization of D-xylose, since lignocellulosic feedstocks contain a significant amount of this pentose sugar [3]. In industrial fermentation processes, the yeast *Saccharomyces cerevisiae* is generally used for bioethanol production. Since *S. cerevisiae* cannot naturally ferment pentose sugars like D-xylose, it has been converted into a xylose-fermenting yeast via the introduction of a fungal xylose isomerase [4,5] (Figure 1). Although this results in the desired D-xylose fermentation, the consumption of D-xylose in the presence of a high D-glucose concentration remains difficult [6]. In general, xylose-fermenting *S. cerevisiae* strains first consume the D-glucose, before D-xylose is metabolized. In an industrial setting, it is preferred that both sugars are fermented simultaneously and at high rates [7] to generate an economically feasible process. The first hurdle for efficient D-xylose consumption in the presence of D-glucose is the uptake of D-xylose by the yeast cell [8], while subsequent suboptimal intracellular metabolism and redox balancing also hamper the process. Some of these issues have been solved by overexpression of the genes of the pentose phosphate pathway [5] (Figure 1) or by the regulation of the redox state during D-xylose fermentation [9]. Other approaches include the introduction of specific D-xylose transporters derived from other organisms [10,11], but these heterologous systems only support low rates of D-xylose transport [12-15] and are often not well integrated in the endogenous carbon metabolism regulatory network of *S. cerevisiae*. Recently, mutagenesis of the *HXT7* and *GAL2* genes yielded a *GAL2* mutant that was found to be defective in D-glucose uptake while still retaining substantial D-xylose transport activity [16].

Glucose transport in *S. cerevisiae* is mediated by the hexose transporter (Hxt) family of sugar transporters [17,18]. A strain in which the main sugar transporter genes *HXT1-17* and *GAL2* were deleted was found to be unable to grow on D-xylose and D-glucose. In this strain, growth on D-xylose could be restored by the reintroduction of *HXT4*, *HXT5*, *HXT7*, or *GAL2* [6]. Following a similar approach with a deletion strain lacking the main hexose transporters (Hxt1-7 and Gal2), uptake of D-xylose could be restored by the reintroduction of *HXT1* and *HXT2* [19]. A major drawback of these endogenous Hxt transporters is their low affinity for D-xylose as compared to D-glucose, which results in D-glucose being the preferred substrate for uptake in mixed sugar fermentations. Consequently, D-xylose-fermenting yeast strains first utilize D-glucose until depletion before D-xylose is metabolized [6,19].

Here, we have used a specific screening and *in vivo* engineering method of endogenous and expressed Hxt transporters in a xylose-fermenting *S. cerevisiae* strain in order to solve the sequential sugar uptake predicament. Using a quadruple hexokinase mutant unable to grow on glucose, an evolved strain was obtained that can grow on D-xylose in the presence of very high concentrations of D-glucose. Growth could be attributed to a mutated endogenous Hxt36 transporter, which has lost the ability to transport D-glucose, while gaining a higher affinity for D-xylose. Further investigation of the sequence space of the mutated amino acid position in Hxt36 allowed us to re-engineer the D-glucose transporter into a specific D-xylose transporter with good transport kinetics. This mutant transporter enables the co-consumption of D-glucose and D-xylose in mixed sugar fermentation.

## Results

### Evolutionary engineering of a xylose-metabolizing *S. cerevisiae* strain in the presence of glucose

**Figure 2:**
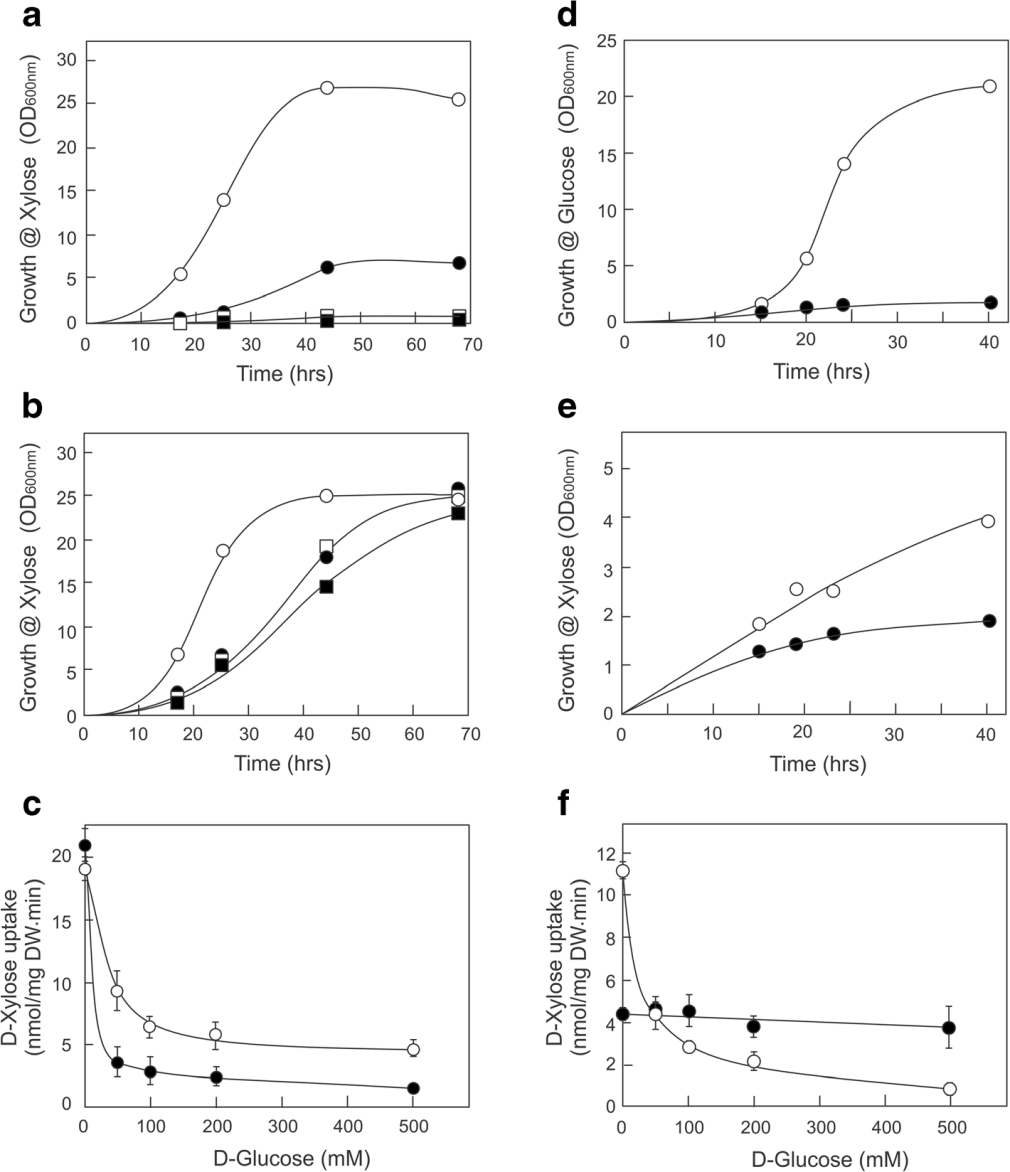
**D-glucose-insensitive uptake of D-xylose by engineered** ***S. cerevisiae*** **strains.** Growth of strain DS71054 **(a)** and DS71054 EvoB **(b)** on 1% D-xylose and varying D-glucose concentration: 0% (open circles), 3% (solid circles), 6% (open squares), and 10% (solid squares). **(c)** Uptake of 50 mM [^14^C-] D-xylose by the DS71054 (open circles) and DS71054 EvoB (solid circles) strains in the presence of competing concentrations of D-glucose. Growth of the DS68625 strain expressing *HXT36* (open circles) or *HXT36*-N367I (solid circles) on 2% D-glucose **(d)** or 2% D-xylose/0.05% maltose **(e)**. Growth on xylose was corrected for the slight background growth on 0.05% maltose using the DS68625 strain transformed with an empty plasmid. **(f)** Uptake of 50 mM [^14^C-] D-xylose by the DS68625 strain expressing *HXT36* (open circles) or *HXT36*-N367I (solid circles) in the presence of various glucose concentrations. Strain DS71054 contains a xylose-fermenting pathway and lacks the hexose kinases necessary for growth on glucose, but contains a full complement of hexose transporters. This strain grows on xylose but can no longer grow on glucose. Strain DS68625 contains a xylose-fermenting pathway but lacks the *HXT1-7* and *GAL2* genes and therefore cannot grow on xylose, while it grows poorly on glucose.

To select for an improved D-xylose transport in *S. cerevisiae*, evolutionary engineering was performed with the quadruple hexokinase (*GLK1, HXK1, HXK2*, and *GAL1*) deletion strain *S. cerevisiae* DS71054, which is described in detail elsewhere (Shin *et al.*, submitted). The DS71054 strain is derived from DS71055, which contains an engineered D-xylose metabolic pathway (Figure 1), and thus is capable of growing on D-xylose [4,20,21] (Figure 2a and Additional file 1: Figure S1) but does not grow on D-glucose (Additional file 1: Figure S1). The strain was used in an evolutionary design experiment to isolate higher affinity D-xylose transporters that are insensitive to D-glucose inhibition. Herein, cells were grown in batch culture on D-xylose (1 to 0.57%), in the presence of increasing concentrations of D-glucose (3 to 10%). Because of the experimental setup, the strain consumes D-xylose but not D-glucose, and this leads to higher D-glucose to D-xylose ratios in time and consequently in a reduction of the growth rate, measured as a decrease in CO_2_ production during the fermentation. However, the specific growth rate on D-xylose in the presence of D-glucose increased during the experiment because of adaptation eventually allowing cells (DS71054 EvoB) to grow on D-xylose (0.57%) in the presence of a 17.5-fold excess of D-glucose (10%). The DS71054 EvoB strain was analyzed for growth in shake flasks on 1% D-xylose in the presence of various concentrations of D-glucose and compared to the progenitor DS71054. Growth of the DS71054 strain was already inhibited at 3% D-glucose and absent at a D-glucose concentration of 6% or higher (Figure 2a). In contrast, growth of the DS71054 EvoB strain on D-xylose was insensitive to high concentrations of D-glucose (Figure 2b). Control experiments demonstrated that the strain is unable to consume D-glucose, nor does it grow on D-glucose (Additional file 1: Figure S1).

To examine the molecular basis that allows the D-glucose-insensitive growth of the DS71054 EvoB strain on D-xylose, D-xylose and D-glucose transport experiments were carried out. In the absence of D-glucose, the DS71054 progenitor and the DS71054 EvoB strain showed similar rates of D-xylose transport (Figure 2c). In the presence of a tenfold excess of D-glucose, D-xylose transport by the DS71054 strain was completely abolished, while for the DS71054 EvoB strain most of the D-xylose uptake was inhibited except for a significant residual rate of D-xylose transport that remained even at very high D-glucose concentrations (Figure 2c). This suggests that the evolution experiment resulted in an evolved strain that shows D-xylose transport with a decreased D-glucose sensitivity.

### A mutated chimeric Hxt36 transporter is responsible for D-glucose-insensitive D-xylose transport in the DS71054 EvoB strain

The DS71054 strain contains the full complement of Hxt transporters, and thus the glucose-insensitive residual xylose uptake by the DS71054 EvoB strain must result from one or more endogenous Hxt transporters. To identify the transporter(s) with altered glucose sensitivity, the expression levels of *HXT1-17* and *GAL2* in the evolved DS71054 EvoB were compared with the original DS71054 strain (Additional file 1: Figure S2). Cells were grown in shake flasks in minimal medium containing 1% xylose and 3% or 10% glucose. The *HXT* gene expression in the evolved DS71054 EvoB strain was similar to that of the progenitor strain (Additional file 1: Figure S2), suggesting that the evolved phenotype is not due to an altered expression of one or more of the Hxt transporters. The altered *HXT* gene expression in the DS71054 EvoB grown with 1% xylose and 10% glucose is due to the high glucose concentration that causes the overexpression of *HXT1* and the down-regulation of *HXT2* and *HXT7* [22]. Based on the absolute C(t) values, only the *HXT1-7* genes are expressed at intermediate or high levels, while *HXT36* showed the highest expression level (data not shown). In the DS71054 strain *HXT3* and *HXT6* are fused, in a similar way as described for *HXT6* and *HXT7* [23]. Because of the high homology between these genes, a chimeric *HXT36* gene was obtained, of which the first 438 amino acids are of *HXT3* and the last 130 of *HXT6* [23]. The Hxt36 chimera is a functional D-glucose transporter (Figure 2e). In the original CEN.PK progenitor strain, these two genes are tandemly arranged in the genome.

Since the expression levels did not identify clear candidates, the genes of the Hxt1-7 and Gal2 transporters were amplified from cDNA isolated from the DS71054 EvoB and progenitor strains and sequenced. This revealed no mutations in *HXT1, HXT2*, and *HXT4*, one silent mutation in *HXT5* (T924C) and *HXT7* (T834A), and an A1100T mutation causing a N367I amino acid substitution in the chimeric *HXT36* gene. The N367I point mutation is located in transmembrane domain (TMD) 8 of Hxt36, which is a region in the Hxt family of transporters known to contain residues involved in D-glucose binding [24]. N367 in Hxt36 corresponds to the same residues in Gal*2* (N376) and Hxt7 (N370) that recently have been shown to be involved in determining the D-glucose transport affinity [16].

The genes encoding *HXT36* and *HXT36*-N367I were amplified from cDNA isolated from DS71054 and DS71054 EvoB cells, respectively, and cloned into the expression plasmid pRS313-P7T7 under control of the Hxt7 promoter. The respective plasmids were transformed to *S. cerevisiae* strain DS68625, in which the *HXT1-7* and *GAL*2 genes are deleted. Because of this transporter deficiency, this strain is unable to grow on D-xylose and is severely defective in its growth on D-glucose. The respective DS68625 strains were pre-grown on 2% maltose and transferred to minimal medium containing 2% D-glucose or D-xylose. The DS68625-Hxt36 strain shows efficient growth on D-glucose. In contrast, growth of the DS68625-Hxt36-N367I mutant strain on D-glucose was almost completely abolished (Figure 2d), whereas both strains still grow on D-xylose (Figure 2e) albeit inefficiently, likely because growth on xylose now solely depends on the Hxt36 transporter. These data suggest that the Hxt36-N367I mutant has lost the ability to transport D-glucose. To examine the suspected specificity change by the N367I mutation, the ability of D-glucose to compete for [^14^C-] D-xylose uptake was examined. At 50 mM, D-xylose uptake by the Hxt36 transporter was strongly inhibited by an equimolar concentration of D-glucose and completely abolished by a tenfold excess of D-glucose. Remarkably, with the Hxt36-N367I transporter, D-xylose transport was essentially unaffected by a tenfold excess of D-glucose (Figure 2f). These data demonstrate that the N367I mutation in Hxt36 is responsible for the D-glucose resistant growth of the DS71054 EvoB strain on D-xylose.

### N367 in Hxt36 is a critical determinant in sugar specificity

**Figure 3:**
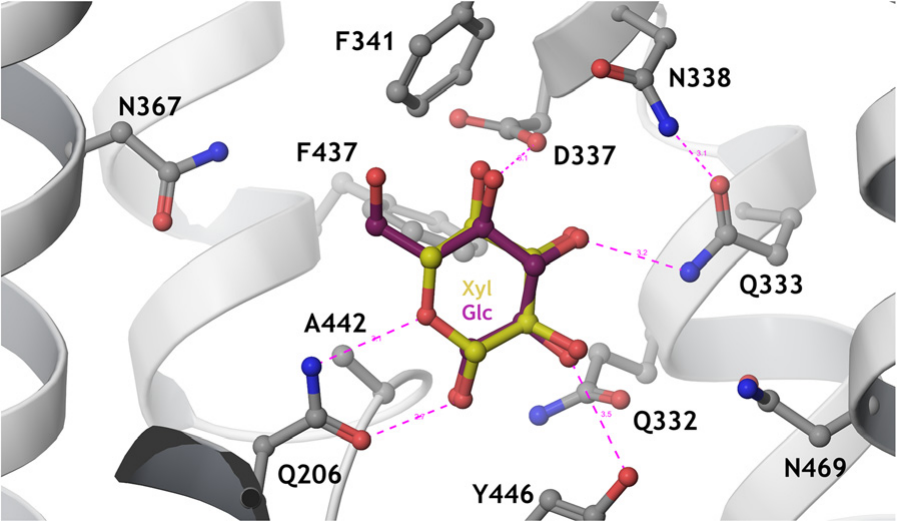
**Detailed view of the sugar-binding pocket of the Hxt36 homology model, showing the first shell amino acid side chains that interact with bound glucose (cyan) and xylose (yellow).** N367 is located to the left, pointing the side chain towards the 6-OH and 6-CH_2_ of glucose. Most residues in this pocket are strictly conserved between Hxt36 and XylE, apart from D337 (I in XylE), A442 (G in XylE), Y446 (W in XylE), and N469 (Q in XylE). The figure was constructed using Maestro (Schrodinger LLC, NY, USA).

The above data show that N367 of Hxt36 is a critical residue that influences the specificity of this hexose transporter. To understand the molecular mechanism of the observed loss of affinity for D-glucose by the N367I mutation, a homology model for Hxt36 was constructed, using the crystal structure of XylE from *Escherichia coli* with xylose bound (PDB ID: 4GBY) [25] as a modeling template. Notably, the region in TMD 8 is conserved between Hxt36 and XylE as residues 364-GVV**N**-367 of Hxt36 are replaced by 322-GVI**N**-325 in XylE, where the conserved asparagine residue is marked in boldface (Figure 3). Therefore, as previously observed for N325 in the crystal structure of XylE, our model indicates that N367 in Hxt36 is in similar close proximity to the 5-CH_2_ of the ring form of D-xylose. In the case of a bound D-glucose in Hxt36 (Figure 3), the 6-CH_2_ and 6-OH would point towards N367, which upon mutation to a hydrophobic isoleucine apparently prevents the D-glucose from binding, while allowing D-xylose to still bind unobstructed.

**Figure 4:**
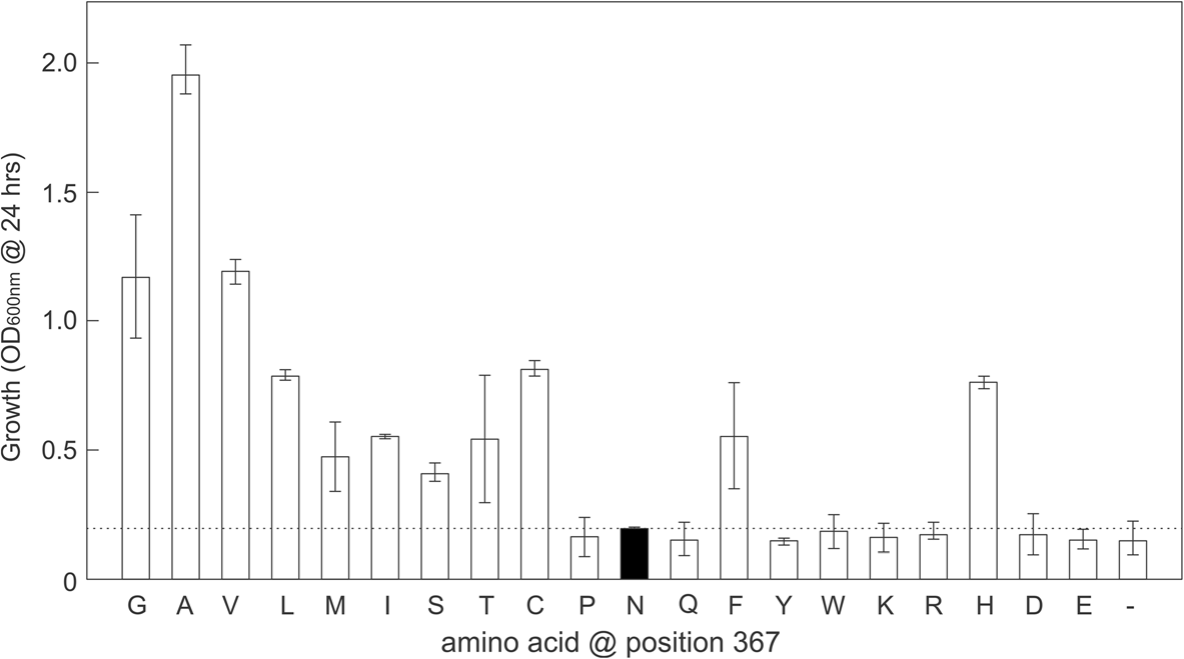
**Growth of the DS71054 strain containing vectors expressing Hxt36 transporters with all possible amino acid substitutions at position 367.** Cells were grown on 1% D-xylose and 10% D-glucose, and DS71054 transformed with the empty vector pRS313-P7T7 was used as a control.

To explore the sequence space of position N367, all amino acid substitutions were introduced individually into the *HXT36* gene. The Hxt36-N367X mutants were transformed to the DS71054 hexokinase deletion strain and tested for growth on minimal medium containing 1% D-xylose and 10% D-glucose. The Hxt36-N367I mutant showed an OD_600_ of 0.56 after 24 h of growth, whereas the wild-type Hxt36 was unable to grow under these conditions (Figure 4). The fastest growing mutant was Hxt36-N367A, which reached an OD_600_ of almost 2 after 24 h. Fast growth was also observed with other nonpolar aliphatic amino acid substitutions (glycine, valine, leucine, and methionine). On the other hand, the phenylalanine and histidine mutants showed a reduced growth rate, whereas amino acid substitutions with strong polar or charged properties did not support growth (Figure 4). The latter could be caused by a catalytic effect or be due to decreased expression, but they were not further examined in order to focus on the improved mutants. These data show that the N367 is a critical residue in determining the specificity of Hxt36 for D-glucose versus D-xylose.

The Hxt36-N367A and N367I mutants were further analyzed to determine their transporter kinetics. Herein, the transporters were expressed in the hexose transport-negative strain DS68625. The K_m_ and V_max_ values for D-glucose uptake by Hxt36 were about 6 mM and 61 nmol/mgDW.min, respectively (see Table 1 and Additional file 1: Figure S3). The Hxt36-N367I mutant was completely defective in D-glucose uptake, while its affinity for D-xylose uptake was improved 2.7-fold (that is, from 108 to 40 mM) as compared to Hxt36 (Table 1). However, the N367I mutation also caused an almost threefold decrease in the V_max_ for D-xylose uptake, which explains the poorer growth on xylose alone (Figure 2e) compared to Hxt36. Moreover, this V_max_ value for xylose is about twofold lower compared to other Hxt transporters such as Hxt7, that however lack this observed specificity towards D-xylose [16]. To examine if the lower V_max_ is due to a decreased expression, chimeras were constructed harboring green fluorescent protein (GFP) fused to the C-terminus of the Hxt36 and the Hxt36-N367I mutant and expressed in the DS68625 strain. Fluorescence imaging revealed that the proteins are uniformly distributed over the plasma membrane (Additional file 1: Figure S4) with nearly identical GFP fluorescence levels, indicating that Hxt36 and Hxt36-N367I are expressed to similar extents. The Hxt36-N367A mutant that showed the fastest growth on D-xylose still mediated some D-glucose uptake although with a very poor K_m_ (171 mM instead of 6 mM). Compared to the N367I mutant, the N367A mutation caused an improvement to both the K_m_ and V_max_ values for D-xylose uptake to 25 mM and 29 nmol/mg DW.min, respectively. This improvement results in better growth of the DS71054 strain harboring Hxt36-N367A compared to the Hxt36-N367I or the Hxt36 wild type when grown on xylose in the presence of excess glucose (Figure 4).

**Table 1:** **K**_**m**_ **and V**_**max**_ **values for D-glucose and D-xylose uptake by Hxt36 transporters expressed in strain DS68625**

	**K**_**m**_ **(mM)**		**K**_**m**_ **ratio**	**V**_**max**_ **(nmol/mg DW.min)**	
	**Glucose**	**Xylose**	**Glc/Xyl**	**Glucose**	**Xylose**
Hxt36	6.1 ± 0.1	107.9 ± 12.1	0.057	60.2 ± 2.0	62.5 ± 5.9
Hxt36-N367I	-^a^	39.8 ± 5.6	-	-^a^	23.0 ± 3.0
Hxt36-N367A	170.7 ± 37.8	24.9 ± 3.4	6.855	70.7 ± 8.4	29.1 ± 0.4

^a^No uptake. Errors are the standard of the mean of three independent experiments.

### Co-consumption of D-glucose and D-xylose by an engineered *S. cerevisiae* strain with altered transport characteristics

**Figure 5:**
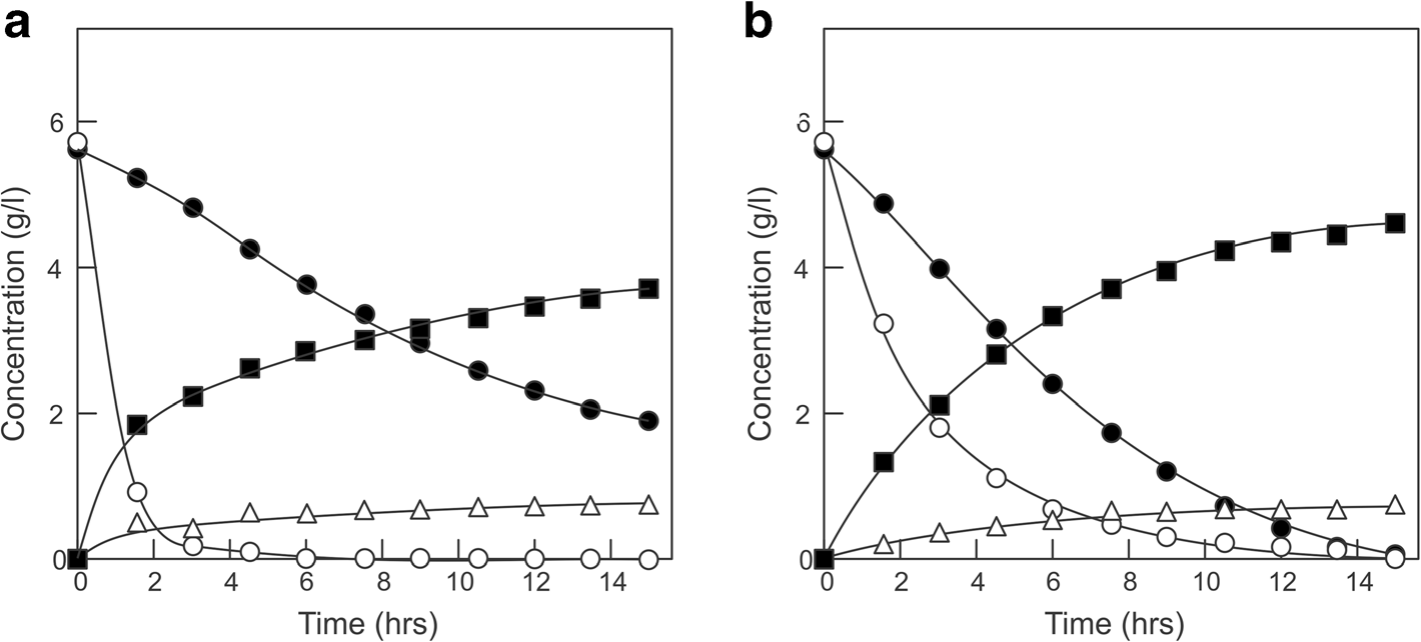
**Growth of the DS68625 strain expressing (a)** ***HXT36*** **, and (b)** ***HXT36*** **-N367A on 0.5% D-glucose and 0.5% D-xylose.** The residual D-glucose (open circles), residual D-xylose (solid circles), ethanol (solid squares), and glycerol (open triangles) were measured in g/l.

To investigate the co-consumption of D-glucose and D-xylose, DS68625 strains harboring wild-type Hxt36, Hxt36-N367I, and Hxt36-N367A were grown on 0.5 g/l D-glucose and 0.5 g/l D-xylose at a higher industrial starting OD_600_ of 8.0 under anaerobic conditions (Figure 5). The strain containing the wild-type Hxt36 transporter showed a rapid consumption of the D-glucose, while D-xylose consumption was delayed, ensuing only once the D-glucose was completely exhausted (Figure 5a). The strain with the Hxt36-N367I transporter grows on D-xylose, but because of the severe D-glucose uptake defect, only background levels of D-glucose consumption occur (Additional file 1: Figure S5) to the level observed for the original DS68625 strain without any reintroduced transporter (data not shown). The strain harboring the Hxt36-N367A mutant showed a marked improved D-glucose and D-xylose co-consumption (Figure 5b, and Additional file 1: Figure S6) as compared to the strain containing the Hxt36 wild-type transporter (Figure 5a) with the concomitant production of ethanol. Furthermore, equal amounts of glycerol were produced in the Hxt36 wild-type transporter and the N367A mutant (compare Figure 5a and b), while no significant amounts of xylitol and acetic acid could be detected. Although the D-glucose consumption in the Hxt36-N367A strain is slower than that of the wild-type, co-consumption of D-glucose and D-xylose results in a more rapid exhaustion of the sugars with enhanced levels of ethanol production. With the Hxt36-N367A strain the D-glucose consumption rate (Q_glc_) is decreased to 0.93 ± 0.01 g G-glc/gDW.h compared to that of the Hxt36 wild type (1.82 ± 0.05 g G-glc/gDW.h). However, the D-xylose consumption rate (Q_xyl_) was increased to 0.27 ± 0.01 g G-xyl/gDW.h in the N367A mutant compared to the low Q_xyl_ of 0.15 ± 0.01 g G-xyl/gDW.h in the wild type (Additional file 1: Table S5). Both mutants (N367I and N367A) show increased conversion rates (0.43 ± 0.01 and 0.41 ± 0.01 gEtOH/g sugar, respectively) compared with that of the Hxt36 wild type (0.39 ± 0.01 gEtOH/g sugar). Furthermore, the ethanol production rate of the Hxt36-N367A mutant strain was improved almost throughout the whole fermentation with the exception of the early growth phase, where the wild-type Hxt36 consumes D-glucose more rapidly (Additional file 1: Figure S6 and Table S6). These data demonstrate the effective co-consumption of D-xylose and D-glucose in a transporter engineered *S. cerevisiae* strain.

## Discussion

Hxt transporters (*HXT1-7*) of the yeast *Saccharomyces cerevisiae* function as facilitators for D-glucose uptake, allowing the cells to grow efficiently on media containing high concentrations of this sugar. The introduction of a D-xylose metabolic pathway in industrial *S. cerevisiae* strains for applications in lignocellulose conversion into bioethanol also necessitates a solution for efficient D-xylose uptake. In an economically viable process, D-glucose and D-xylose need to be co-consumed to reduce the fermentation time, to increase the bioethanol production rate, and to add to the robustness of the fermentation process. Although the *S. cerevisiae* strain DS68625 used in this study metabolizes D-xylose efficiently, the process is strongly inhibited by D-glucose because the uptake of D-xylose occurs via the endogenous hexose transporters which prefer D-glucose instead of D-xylose. To overcome glucose inhibition and to select for variants with a more specific D-xylose uptake, a screening method was devised based on a strain constructed from the D-xylose-metabolizing progenitor DS71055 but lacking the four hexokinase genes. The resultant DS71054 strain cannot grow on D-glucose, but still grows on D-xylose using the endogenous Hxt transporters for uptake. The *in vivo* evolution experiment that selected for improved D-xylose growth in the presence of increasing amounts of glucose yielded the DS71054 EvoB strain that finally grew on D-xylose in the presence of a 17.5-fold excess of D-glucose. Expression, sequencing, and functional analyses demonstrated that a single N367I mutation in the chimeric Hxt36 transporter is responsible for this glucose insensitivity. Remarkably, the N367I mutation completely abolishes D-glucose uptake and allows D-xylose uptake with an improved affinity. However, we also observed a reduction in the V_max_ for D-xylose transport, and consequently the strain carrying the *HXT36*-N367I mutant gene shows a reduced growth on D-xylose as compared to the strain harboring the wild-type *HXT36* gene. Saturation mutagenesis of N367 yielded the Hxt36-N367A mutant that showed a higher V_max_ and a further improved K_m_ for D-xylose as compared to the initial N367I mutant. This mutation does not abolish D-glucose uptake, but causes a dramatic reduction in the D-glucose affinity and thus also a reduced inhibition of D-xylose transporter by D-glucose. The evolutionary engineering of the DS71054 strain did not yield an alanine at position 367, presumably as this required multiple point mutations to change the codon whereas only a single point mutation is needed to convert asparagine into the nonpolar isoleucine. Recently, both heterologous and endogenous glucose transporters were rewired for D-xylose transport in *S. cerevisiae* by saturation and rational mutagenesis of a conserved sequence motif in the first TM domain [26]. Although mutants were obtained that showed selective uptake of D-xylose, none of these mutants was resistant to D-glucose inhibition. In another study, Farwick *et al.* [16] expressed mutants of the *HXT7* and *GAL2* genes in an *hxt0* background to select for D-glucose-insensitive growth on D-xylose. Thus, that study was restricted to a selected number of hexose transporters, whereas our evolutionary engineering of a quadruple hexokinase mutant selects from the full arsenal of relevant hexose transporters. This yielded the Hxt36-variant as a spontaneous high profile candidate for D-xylose transporter under high glucose concentrations, conditions that are relevant for industrial batch fermentations. Since less stringent conditions were used for selection by Farwick *et al.* [16], that is, growth on D-xylose in the presence of a 5-fold instead of a 17.5-fold excess of D-glucose, the resulting specificity mutants exhibit weaker characteristics. The mutations in N376 and N370 for Gal2 and Hxt7, respectively, correspond to the same hot spot N367 in Hxt36. Although the Gal2 N376F mutant was found to be completely defective in D-glucose uptake, it retained only about one-third of the D-xylose uptake activity with a twofold improved K_m_ for D-xylose. This mutant showed properties somewhat similar to those of the Hxt36-N367I mutant presented in this study. However, the phenotype of the Gal2 N376V and the Hxt7 mutants was less distinct. Although a reduction in the K_m_ for D-glucose was achieved, the K_m_ was still an order of magnitude better than for D-xylose, while the V_max_ for D-glucose uptake even increased up to twofold. In these mutants, hardly an improvement in the K_m_ for D-xylose uptake was obtained, whereas a severe loss in activity (more than threefold) was noted. This contrasts the N367A mutation in Hxt36, which causes a more than two orders of magnitude improvement in the affinity of Hxt36 for D-xylose compared to D-glucose, rendering the Hxt36-N367A transporter specific for D-xylose, while the loss in V_max_ was less than twofold.

The asparagine at position 367 is conserved in all Hxt transporters in *S. cerevisiae* but also in functional homologues like the mammalian Glut1 and *E. coli* XylE (homology of 23 and 26%, respectively). This residue is located in TMD 8, which is considered to be important for the D-glucose affinity [24]. In XylE, the asparagine is located at position 325 in the xylose binding site. Although we cannot be certain about the mechanism for the largely reduced affinity of the N367I mutant for D-glucose based only on a model of the occluded substrate bound state of Hxt36, we hypothesize that the hydrophobic isoleucine side chain may prevent the D-glucose from binding in the outward-open state of the transporter because of the lack of proper hydrogen bonding involving the D-glucose 6-OH. On the other hand, D-xylose still binds, and the mutation even caused an increase in the binding affinity. In this respect, the 6-OH and 6-CH_2_ atoms are missing in D-xylose. In general, it appears that the polarity and size of the amino acid side chain at position 367 are important determinants in the sugar specificity. With almost all nonpolar amino acids, except for tryptophan and proline, excellent selectivity for D-xylose over D-glucose has been obtained. The best selectivity appears to be with the small and nonpolar amino acids alanine and glycine. A polar or charged bulky amino acid side chain does not seem to cause selectivity. Interestingly, the N325A mutant of XylE is also still able to transport xylose [25], consistent with our observations.

Although improved selectivity for D-xylose is readily obtained by single mutations, substitutions at position 367 are accompanied with a more or less severe loss in activity. Likely, substitutions at N367 also cause conformational defects in the transporter. Loss of activity is undesirable for the development of an efficient lignocellulose conversion process, but the Hxt36-N367A mutant is still sufficiently active to exhibit co-consumption of D-glucose and D-xylose in mixed sugar fermentations, leading to an overall higher bioethanol production rate. Importantly, the N367A mutation in Hxt36 enables the cells to co-consume D-glucose and D-xylose, likely because it improved the K_m_ ratio for xylose over glucose (Table 1) while still maintaining a good V_max_. This could not be achieved with the Gal2-N367V and Hxt7-N370S mutants, and this may explain why these mutants were not further tested for the co-consumption of D-xylose and D-glucose [16]. Our data demonstrate that, for co-consumption, the rates of D-glucose and D-xylose uptake should be balanced. In the present case, this was realized with only one engineered transporter, but for a more robust fermentation, this balance should be maintained throughout the entire fermentation process with high uptake velocity.

## Conclusions

We have demonstrated the successful conversion of a yeast hexose transporter into a specific pentose transporter or transporters with a tunable specificity for D-xylose and D-glucose. The latter engineered transporter allows for the co-consumption of pentose and hexose sugars by a xylose-fermenting strain. This novel approach of endogenous transporter engineering also has the added benefit that it does not interfere with the post-translational regulatory phenomena in the cell that induce the D-glucose concentration dependent turnover of transporters, thus providing a sustainable solution for bioethanol production.

## Methods

### Molecular biology techniques and chemicals

Restriction enzymes and T4 DNA ligase were acquired from Fermentas - Thermo Fisher Scientific, Pittsburgh, PA, USA. Antibiotics hygromycin (HG), phleomycin (Phleo), and geneticin (G418) were acquired from InvivoGen (San Diego, CA, USA). pYL16 and nourseothricin (nour) were acquired from Werner BioAgents (Jena, Germany) using concentrations recommended by the supplier for selection of the respective antibiotic resistance marker-bearing *S. cerevisiae* transformants. Ampicillin and kanamycin were acquired from Sigma-Aldrich (Zwijndrecht, The Netherlands). Oligonucleotides used for strain constructions were purchased from Sigma-Aldrich. Yeast genomic DNA was isolated from yeast using the YeaStar™ Genomic DNA Kit (Zymo Research, Irvine, CA, USA) following the manufacturer’s instructions.

### Strains and growth conditions

The *S. cerevisiae* strains used in this study (Table S1) were provided by DSM Bio-based Products & Services and are described elsewhere (Shin *et al.*, submitted). Pentose-fermenting *S. cerevisiae* strains were provided by DSM and may be made available for academic research under a strict Material Transfer Agreement with DSM (contact: paul.waal-de@dsm.com). Fed-batch and chemostat cultures (Applikon, Schiedam, The Netherlands) were grown in minimal medium supplemented with vitamin solution and trace elements [27] in a 500-ml working-volume laboratory fermentor at a temperature of 30°C and pH 4.5. The starting dissolved oxygen (DO) set point was 5%, stirring was performed at 400 rpm, and the starting OD_600_ was 0.2. Shake flask experiments at 200 rpm were also done in minimal medium supplemented with 2% D-maltose, 2% D-xylose/0.05% D-maltose, or 0.5% D-xylose/0.5% D-glucose. The 0.05% D-maltose was added in order to circumvent an elongated lag phase in minimal medium with only 2% D-xylose. A starting OD_600_ of 0.1 was used in all experiments except for the anaerobic co-consumption experiment, in which an OD_600_ of 8.0 was used. Cell growth was monitored by optical density (OD) at 600 nm using a UV-visible spectrophotometer (Novaspec Plus).

### RNA extraction and cDNA synthesis

Total RNA was isolated from *S. cerevisiae* cells by a glass-bead disruption Trizol extraction procedure and performed as described by the manufacturer (Life Technologies, Bleiswijk, The Netherlands). Yeast pellets from 2 ml of exponential phase cell culture (OD_600_ of approximately 10) were mixed with 0.2 ml of glass beads (diameter 0.45 mm) and 900 μl of Trizol with 125 μl chloroform, and disrupted in a Fastprep FP120 (Thermo Savant) for 45 seconds at speed 6. The extracted total RNA (1 μg) was used to synthesize cDNA using the iScript cDNA synthesis Kit (Bio-Rad, Hercules, CA, USA).

### Primers and real-time PCR

Real-time PCR on the expression of *HXT1-17* and *GAL2* was performed with the primers indicated in Additional file 1: Table S3, using the SensiMix SYBR & Fluorescein Kit (Quantace Ltd.) and the iCycler iQ Real-Time PCR instrument (Bio-Rad). Actin was used as a reference gene to normalize fold changes. The SYBR Green Master Mix (12.5 μl) was used for 25 μl reactions containing 4 μl of the extracted total RNA (10 ng/μl), 1 μl of the indicated primers (10 nM), and 6.5 μl of sterile water. The PCR conditions for *HXT1-17* and *GAL2* were 10 min at 95°C followed by 40 cycles of amplification (15 s at 95°C, 30 s at 58°C, 30 s at 72°C).

### Sequencing and general cloning

High/intermediate expressed genes were amplified using the primers listed in Additional file 1: Table S2 using the Phusion® High-Fidelity PCR Master Mix with HF Buffer. The full-length cDNA of *HXT36* and *HXT36*-N367I was amplified using primers F HXT36 BcuI and R HXT36 BamHI (Additional file 1: Table S2) employing cDNA isolated from a batch culture of the cells grown on minimal medium containing 1% xylose and 3% glucose. The vector pRS313-P7T7 was used for the expression of HXT transporters under control of the *HXT7* promoter and was derived from pRS313 (kindly supplied by DSM Biotechnology Center, The Netherlands) as a backbone containing the histidine selection marker and the Cen/ARS low copy origin for cloning in yeast. pRS313 was digested with the restriction enzymes SacI and XbaI and Bsu15I and SalI, respectively, and the promoter of *HXT7* (391 bp) and the terminator of *HXT7* (1000 bp) were cloned into the multiple cloning site, yielding pRS313-P7T7. The promoter and terminator were amplified with Phusion® High-Fidelity PCR Master Mix with HF Buffer using the primer pairs F SacI s promHXT7/R promHXT7 XbaI and F terHXT7 Bsu15I/R terHXT7 SalI, respectively (Additional file 1: Table S4).

The saturated mutagenesis of position N367 in HXT36 was done using PCR with Phusion® High-Fidelity PCR Master Mix with HF Buffer using primer pairs F HXT36 BcuI/R HXT36 367NNN and F HXT36 367NNN/R HXT36 BamHI (Additional file 1: Table S4). The fragments of 1119 and 623 base pairs were subsequently used in an overlap PCR using the outside primers F HXT36 BcuI and R HXT36 BamHI and cloned into pRS313-P7T7 using BcuI and BamHI. Sequencing of 48 *E. coli* clones yielded N367S (tcc), N367P (ccc), N367G (ggg), N367Y (tac), N367A (gcc), N367H (cac), N367R (agg), N367F (ttt), N367E (gag), and N367V (gtg). The remaining eight amino acids at position 367 were amplified and cloned as mentioned above with overlap PCR using specific primers in which the NNN was replaced by tta (L), tgt (C), tgg (W), atg (M), act (T), aag (K), gat (D), and cag (Q).

The C-terminal GFP fusions with HXT36 and the HXT36-N367I mutant were made by amplification of the corresponding genes with the Phusion® High-Fidelity PCR Master Mix (HF Buffer) using primers F HXT36 BcuI and R HXT36 BamHI-stop (Additional file 1: Table S4). The GFP gene itself was amplified with F GFP BamHI and R GFP ClaI. *HXT36* and *HXT36*-N367I were digested with the restriction enzymes BcuI and BamHI, and GFP was digested with BamHI and ClaI. The *HXT36* genes were separately ligated in a two-fragment ligation together with GFP into pRS313-P7T7, which was cut with BcuI and ClaI.

### *In vivo* evolution

The quadruple hexokinase deletion mutant DS71054 was evolved in batch cultivation to grow on a relatively low D-xylose concentration (1 to 0.57%) in the presence of increasing concentrations of D-glucose (3 to 10%). Growth was followed in time by CO_2_ measurements, while the levels of D-xylose and D-glucose were monitored to ensure that the cells were growing solely on D-xylose. The glucose-to-xylose ratio at the start of the evolutionary engineering was kept low but increased during the experiment, eventually reaching 0.57% D-xylose and 10% D-glucose. In the setup, the strain consumes the D-xylose, which leads to higher glucose-to-xylose ratios and consequently a drop in growth rate. Once the CO_2_ production was reduced, additional xylose (5 ml of 50% xylose added to the 500-ml fermentor volume) was added to maintain growth, and at regular time intervals (on average after 5 to 6 days), the culture was diluted into fresh medium with a higher glucose-to-xylose ratio. After 27 days, the evolved DS71054 strain was obtained and plated on 1% xylose and 10% glucose.

### Analytical methods

High performance liquid chromatography (Shimadzu, Kyoto, Japan) was performed using an Aminex HPX-87H column at 65°C (Bio-Rad), and a refractive index detector (Shimadzu, Kyoto, Japan) was used to measure the concentrations of D-glucose, D-xylose, and ethanol. The mobile phase was 0.005 N H_2_SO_4_ at a flow rate of 0.55 ml/min.

### Uptake measurement

The sugar uptake was measured as follows: cells were grown for 24 h in shake flasks in minimal medium containing 2% maltose and were collected by centrifugation (3,000 rpm, 3 min, 20°C), washed, and resuspended in minimal medium without a carbon source. [^14^C] xylose or [^14^C] glucose stocks were added to the cell suspension, and the reaction was stopped at various time intervals by the addition of 5 ml of ice cold 0.1 M lithium chloride. The samples were filtered over 0.45-μm HV membrane filters (Millipore, France), washed once with an ice cold solution of 5 ml of lithium chloride, and counted by Liquid Scintillation Counter (PerkinElmer, Waltham, MA, USA). The D-xylose and D-glucose concentrations were varied from 0.5 to 500 mM and 0.1 to 500 mM, respectively. For the competition experiments, the uptake of 50 mM [^14^C-] D-xylose was analyzed in the presence of 50 to 500 mM unlabeled D-glucose.

### Molecular modeling

The molecular modeling software package, including Prime 3.1 and Maestro 9.3, from Schrodinger LLC (New York, NY, USA) was used to build a homology model for Hxt36. The crystal structure of XylE from *E. coli* with xylose bound (PDB ID: 4GBY [25]), which is 26% identical to Hxt36p, was used as a template for the model building. Because of the lower identity, the sequence alignment between Hxt36p and XylE was manually corrected using information from family sequence alignments in the program Prime to accurately reflect the correct location of amino acid insertions and deletions.

### Fluorescence microscopy

Fresh colonies of transformants expressing the *HXT36* and *HXT36*-N367I mutant in DS68625 were inoculated in triplicate in minimal medium with 2% D-maltose and grown to exponential growth phase (at an OD_600_ of approximately 5). To determine the cellular localization, the fluorescence was analyzed using a Nikon Eclipse-Ti microscope equipped with an 100× oil immersion objective, a filter set for GFP, and a Nikon DS-5Mc cooled camera. The total amount of GFP fluorescence was analyzed (at an emission of 507 nm) on a BioTek Synergy Mx 96-well plate reader. The total amount of GFP fluorescence was corrected for the optical density (OD_600_) of the cultures.

## Additional file

Additional file 1: Figure S1. Growth (OD_600_) of the DS71054 strain on 2% D-xylose (solid circles) and 2% D-glucose (open circles). **Figure S2.** Normalized fold expression of *HXT1-17* and *GAL2*. Gene expression was analyzed on minimal medium with 1% xylose and 3% glucose for the DS71054 (white bars) and DS71054 EvoB (gray bars) strains. The gene expression in the DS71054 EvoB strain was also analyzed on 1% D-xylose and 10% D-glucose (black bars). Fold expression was normalized relative to the expression in the DS71054 that was set to 1. **Figure S3.** Kinetic analysis of D-glucose **(A)** and D-xylose **(B)** uptake by the DS68625 strain expressing *HXT36* (open circles), *HXT36*-N367I (open squares), and *HXT36*-N367A (solid squares). Uptake was corrected for the background observed with the DS68625 strain transformed with the empty expression vector and expressed in nmol/mgDW.min. **Figure S4.** Fluorescence images of strain DS68625 expressing GFP fusion proteins of Hxt36 **(A)** and Hxt36-N367I **(B)**. Images were analyzed on a Nikon Eclipse-Ti microscope. **(C)** Total amount of GFP fluorescence (in AU/OD_600_) in both strains as measured in a spectrofluorometer. **Figure S5.** Growth of the DS68625 strain expressing *HXT36*-N367I on 0.5% D-glucose and 0.5% D-xylose. The residual D-glucose (open circles), residual D-xylose (solid circles), ethanol (solid squares), and glycerol (open triangles) were measured in g/l. **Figure S6.** Ethanol production rates of the DS68625 strain expressing *HXT36* (circles), *HXT36*-N367I (squares), and *HXT36*-N367A (triangles) were measured in gEtOH/gDW.h. The indicated standard error represents the mean of two independent experiments. **Table S1.** Strains and plasmids used in this study. **Table S2.** Oligonucleotides used in hexose transporter strain construction. **Table S3.** Oligonucleotides used in qPCR. **Table S4.** Oligonucleotides used in cloning and sequencing. **Table S5.** Sugar conversion parameters of strain DS68625 expressing the indicated Hxt36 transporters when grown on 0.5% D-glucose and 0.5% D-xylose.

## Abbreviations

HK: hexokinase
HXT: hexose transporter
OD: optical density
PCR: polymerase chain reaction
XKS: xylulose kinase
XI: xylose isomerase
