# Supplementary material for: Engineering of an endogenous hexose transporter into a specific D-xylose transporter facilitates glucose-xylose co-consumption in Saccharomyces cerevisiae
Source: Biotechnol Biofuels. 2014 Nov 29;7:168. doi: 10.1186/s13068-014-0168-9 (PMC4263072; doi:10.1186/s13068-014-0168-9)
Supplement: Additional file 1: Figure S1. — Growth (OD600) of the DS71054 strain on 2% D-xylose (solid circles) and 2% D-glucose (open circles). Figure S2. Normalized fold expression of HXT1-17 and GAL2. Gene expression was analyzed on minimal medium with 1% xylose and 3% glucose for the DS71054 (white bars) and DS71054 EvoB (gray bars) strains. The gene expression in the DS71054 EvoB strain was also analyzed on 1% D-xylose and 10% D-glucose (black bars). Fold expression was normalized relative to the expression in the DS71054 that was set to 1. Figure S3. Kinetic analysis of D-glucose (A) and D-xylose (B) uptake by the DS68625 strain expressing HXT36 (open circles), HXT36-N367I (open squares), and HXT36-N367A (solid squares). Uptake was corrected for the background observed with the DS68625 strain transformed with the empty expression vector and expressed in nmol/mgDW.min. Figure S4. Fluorescence images of strain DS68625 expressing GFP fusion proteins of Hxt36 (A) and Hxt36-N367I (B). Images were analyzed on a Nikon Eclipse-Ti microscope. (C) Total amount of GFP fluorescence (in AU/OD600) in both strains as measured in a spectrofluorometer. Figure S5. Growth of the DS68625 strain expressing HXT36-N367I on 0.5% D-glucose and 0.5% D-xylose. The residual D-glucose (open circles), residual D-xylose (solid circles), ethanol (solid squares), and glycerol (open triangles) were measured in g/l. Figure S6. Ethanol production rates of the DS68625 strain expressing HXT36 (circles), HXT36-N367I (squares), and HXT36-N367A (triangles) were measured in gEtOH/gDW.h. The indicated standard error represents the mean of two independent experiments. Table S1. Strains and plasmids used in this study. Table S2. Oligonucleotides used in hexose transporter strain construction. Table S3. Oligonucleotides used in qPCR. Table S4. Oligonucleotides used in cloning and sequencing. Table S5. Sugar conversion parameters of strain DS68625 expressing the indicated Hxt36 transporters when grown on 0.5% D-glucose and 0.5% D-xylose. [file 13068_2014_168_MOESM1_ESM.docx]

**Supplemental data**

**
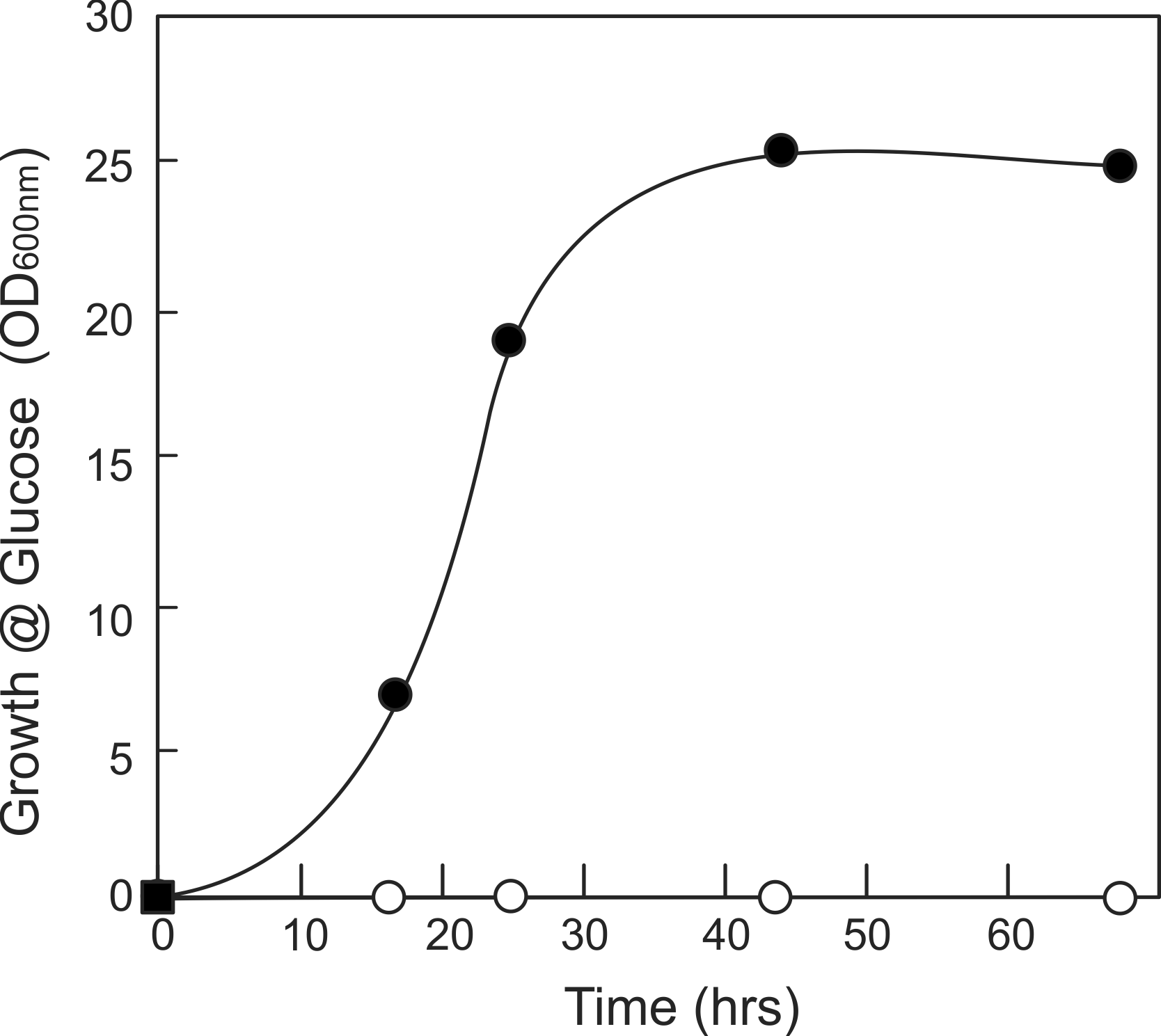
**

**Supplemental Fig. 1.** Growth (OD_600_) of the DS71054 strain on 2 % D-xylose (⚫) and 2 % D-glucose (⭘).

**
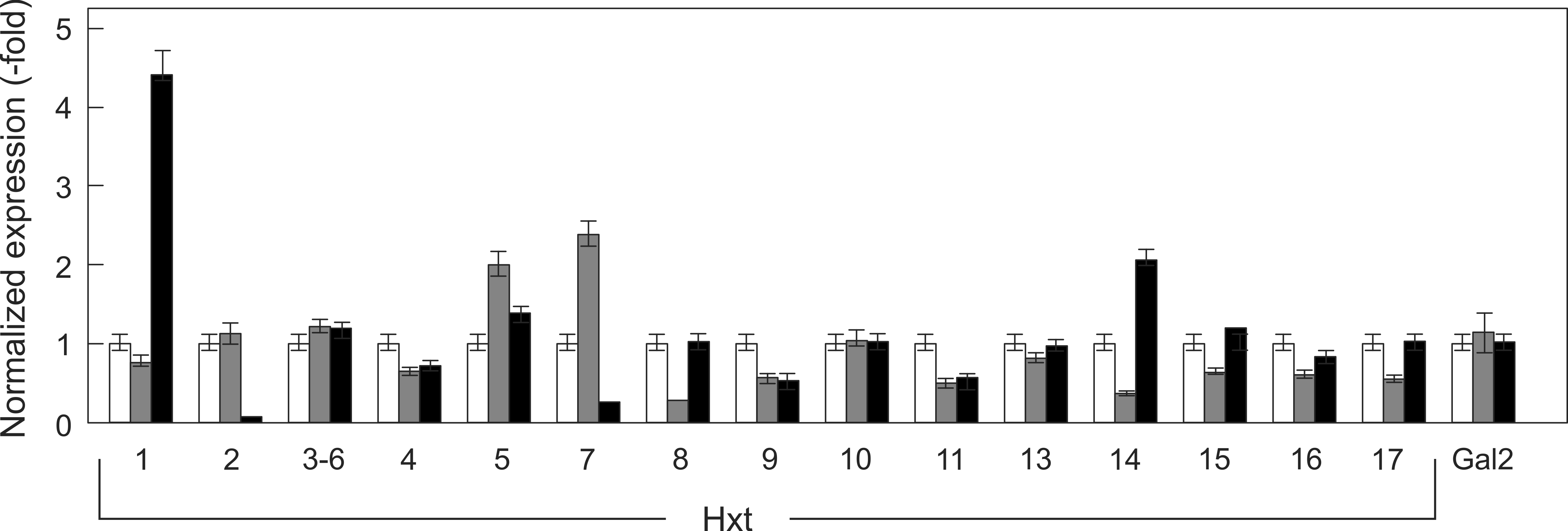
**

**Supplemental Fig. 2.** Normalized fold expression of *HXT1-17* and *GAL2*. Gene expression was analyzed on minimal medium with 1 % xylose and 3 % glucose for the DS71054 (white bars) and DS71054 EvoB (grey bars) strains. The gene expression in the DS71054 EvoB strain was also analyzed on 1 % D-xylose and 10 % D-glucose (black bars). Fold expression was normalized relative to the expression in the DS71054 that was set to 1.


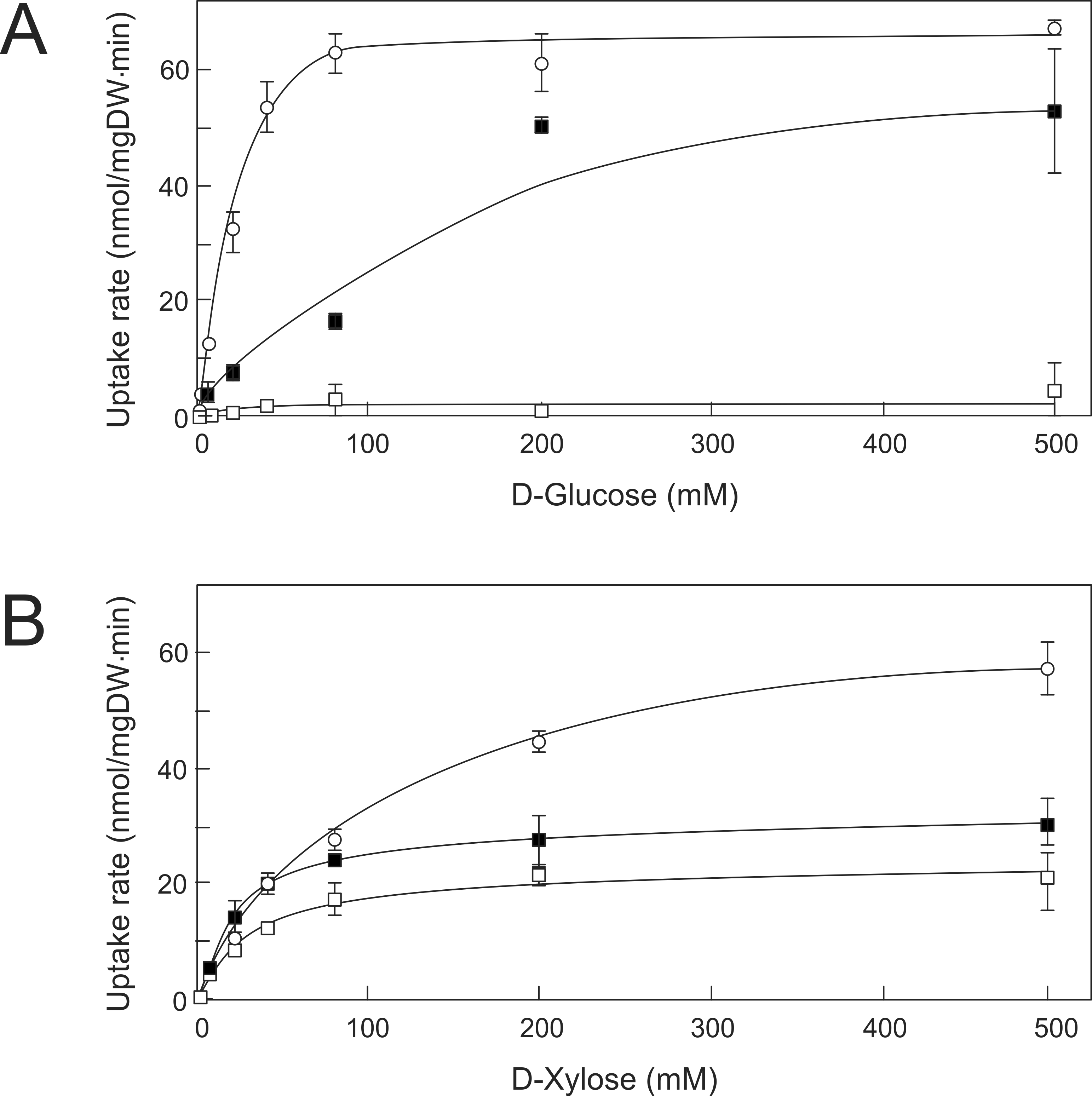


**Supplemental Fig. 3.** Kinetic analysis of D-glucose (**A**) and D-xylose (**B**) uptake by the DS68625 strain expressing *HXT36* (⭘), *HXT36*-N367I (🞏) and *HXT36*-N367A (◼). Uptake was corrected for the background observed with the DS68625 strain transformed with the empty expression vector and expressed in nmol/mgDW.min.

**
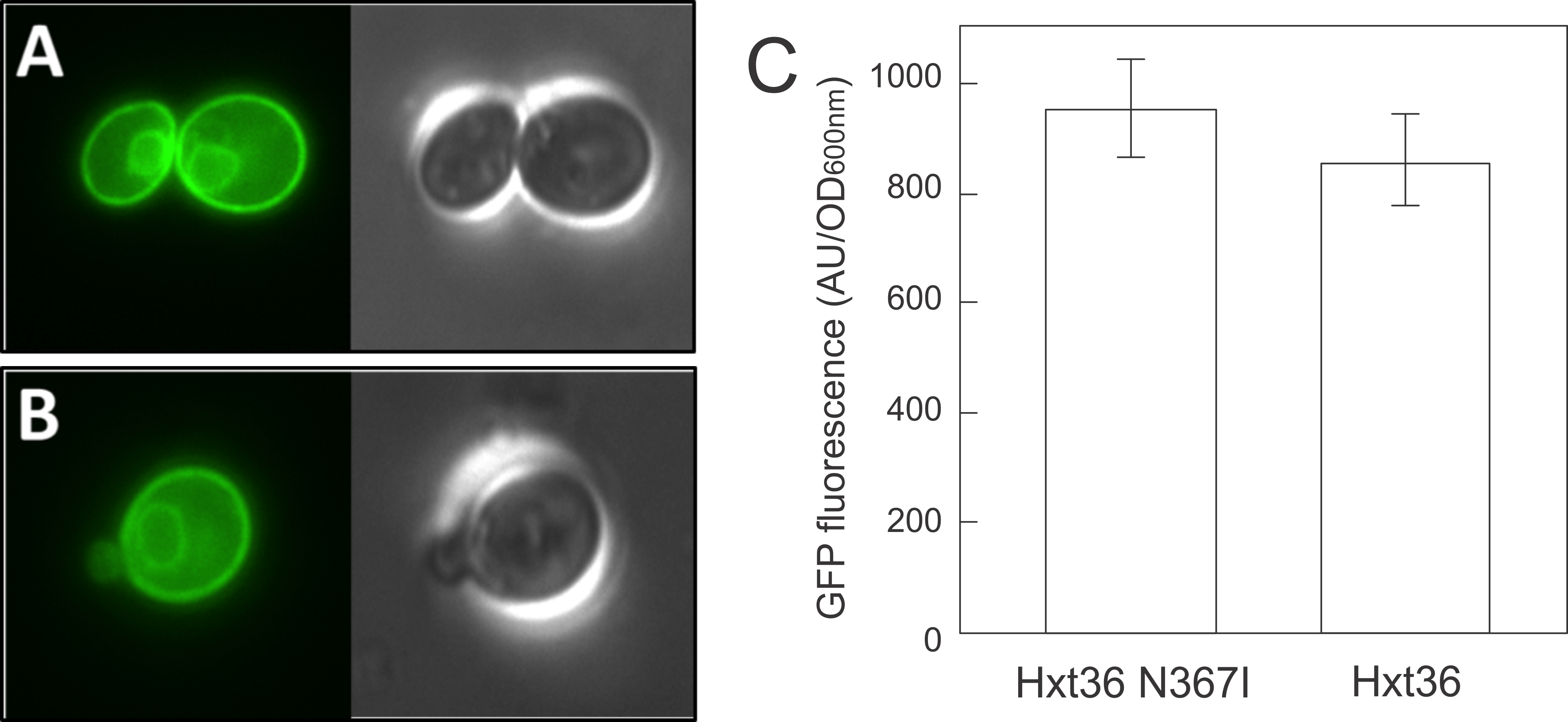
**

**Supplemental Fig. 4.** Fluorescence images of strain DS68625 expressing GFP fusion proteins of Hxt36 (**A**) and Hxt36-N367I (**B**)**.** Images were analyzed on a Nikon Eclipse-Ti microscope. (C) Total amount of GFP fluorescence (in AU/OD_600_) in both strains as measured in a spectrofluorimeter.


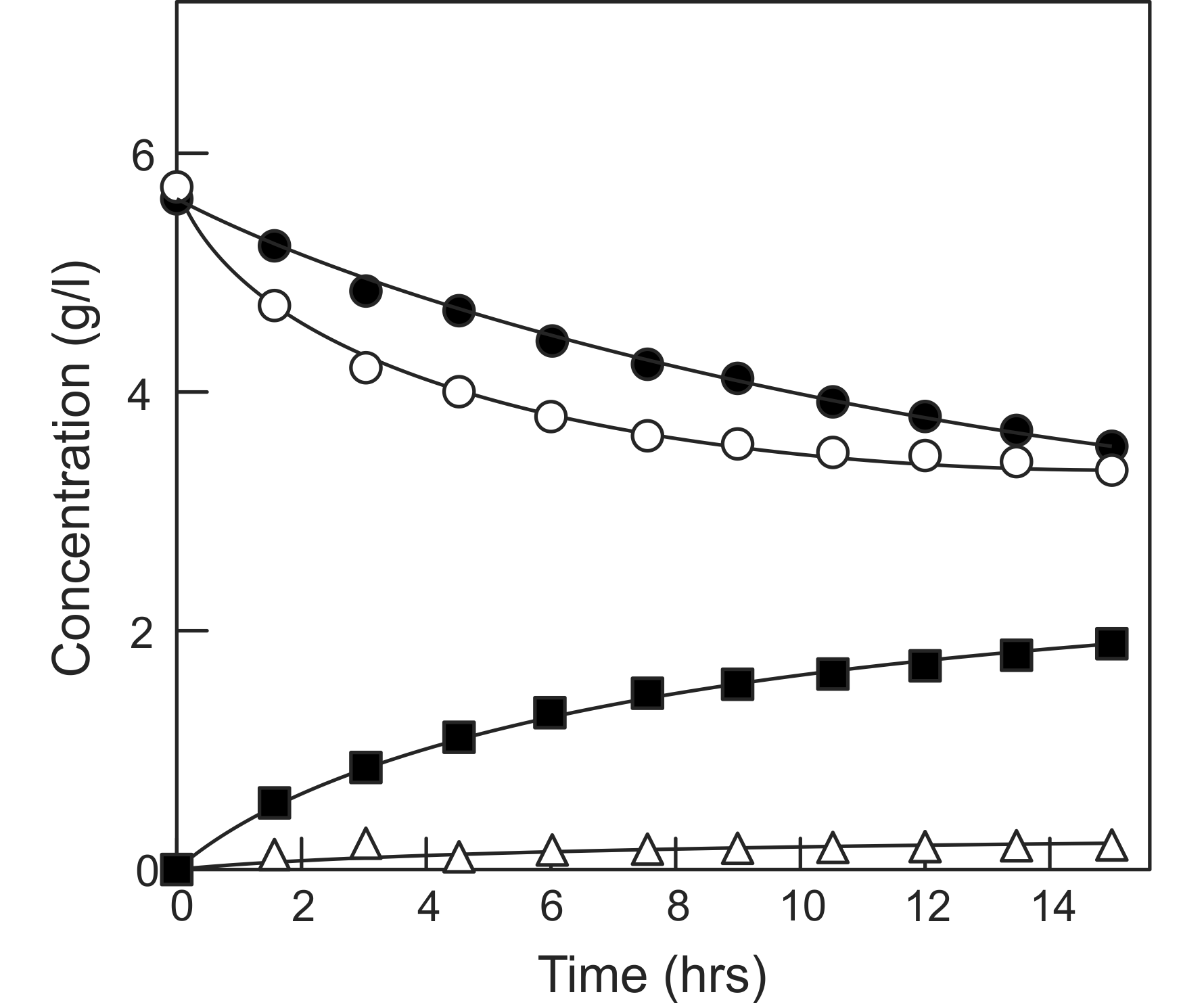


**Supplemental Fig. 5.** Growth of the DS68625 strain expressing HXT36-N367I on 0.5 % D-glucose and 0.5 % D-xylose. The residual D-glucose (⭘), residual D-xylose (⚫), ethanol (◼) and glycerol (△) were measured in g/l.


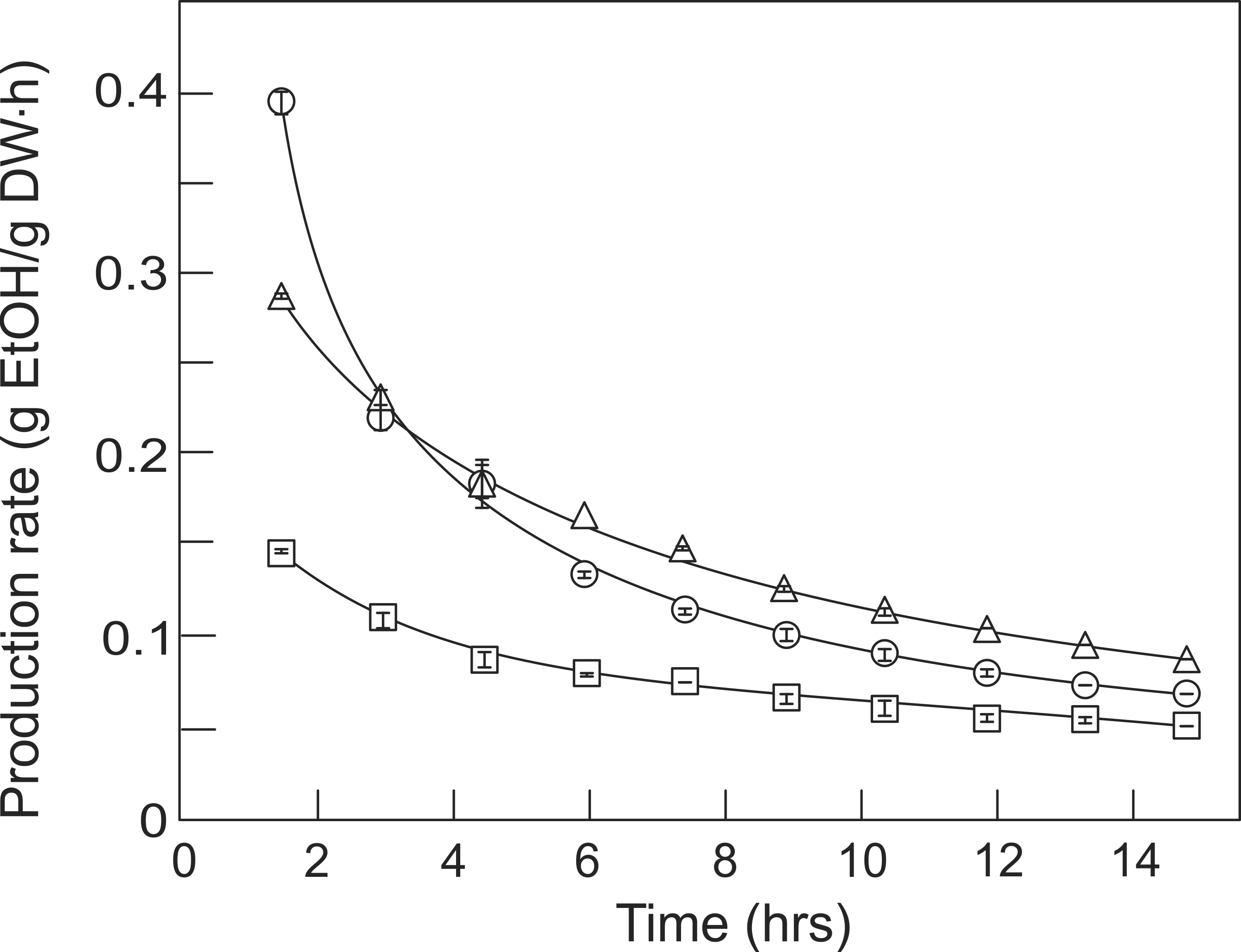


**Supplemental Fig. 6.** Ethanol production rates of the DS68625 strain expressing *HXT36* (⭘), *HXT36*-N367I (□) and *HXT36*-N367A (△) were measured in gEtOH/gDW.h). The indicated standard error represents the mean of two independent experiments.

**Supplemental Table 1. Strains and plasmids used in this study**

| Strain/ plasmid | Relevant genotype and/or characteristics | Source or reference |
| --- | --- | --- |
| Strains |  |  |
| *S. cerevisiae* |  |  |
| DS68616 | *Mat a, ura3-52, leu2-112, gre3::loxP, loxP-Ptpi:TAL1, loxP-Ptpi::RKI1, loxP-Ptpi-TKL1, loxP-Ptpi-RPE1, delta::Padh1XKS1Tcyc1-LEU2, delta::URA3-Ptpi-xylA-Tcyc1* | DSM, The Netherlands |
| DS68625 | DS68616, *his3::loxP, hxt2::loxP-kanMX-loxP, hxt367::loxP-hphMX-loxP, hxt145::loxP-natMX-loxP, gal2::loxP-zeoMX-loxP* | This paper |
| DS71055 | DS68616-derivative after evolutionary engineering | This paper |
| DS71054 | DS71055, *glk1*::*lox*72; *hxk1*::*lox*P; *hxk2:*:*lox*72; *gal1:*:*lox*P; *his3*::loxPnatMXloxP | This paper |
| DS71054-evoB | DS71054-derivative after evolutionary engineering by chemostat cultivation on xylose in presence of glucose | This paper |
| Plasmids |  |  |
| pRS313 | *E. coli*/yeast shuttle vector; *CEN6, ARSH4, HIS3,* Amp^r^ | Sikorski & Hieter, 1989 |

**Supplemental Table 2: oligonucleotides used in hexose transporter strain construction**

| Number | Primer | Sequence (5’🡪 3’) | Gene(s) |
| --- | --- | --- | --- |
| 28 | H3f | TGTACATCCGGAATTCTAGATTGGTGAGCGCTAGGAGTCACTGCC | *HIS3* |
| 29 | H3r | CTCGAGTATTTCACACCGCATATGATCCGTCG | *HIS3* |
| 201 | Hx2uf | GACTAGTACCGGTGTTTTCAAAACCTAGCAACCCC | *HXT2* |
| 202 | Hx2ur | CGTACGCGTCTTCCGGAAGGGTACCATCAGATTTCATTTGACC | *HXT2* |
| 203 | Hx2df | GAAGACACTCGAGACGTCCTTTGTCTGTGAAACCAAGGGC | *HXT2* |
| 204 | Hx2dr | GTCGACGGGCCCTTATGTTGGTCTTGTTTAGTATGGCCG | *HXT2* |
| 205 | Hx3uf | AAGCGGCCGCACTAGTACCGGTGAAACAACTCAATAACGATGTGGGAC | *HXT3* |
| 206 | Hx3ur | ATCCGGACGTCTTCCTCAAGAAATCAGTTTGGGCGACG | *HXT3* |
| 210 | Hx4df | AGAAGACGCTCGAGACGTCCCTTATGGGAAGAAGGTGTTTTGCC | *HXT4* |
| 211 | Hx4dr | ATGGATCCTAGGGGTTCTTGCAGAGTAAACTGCG | *HXT4* |
| 212 | Hx5uf | AAGCGGCCGCACTAGTACATGTGAACTTGAAAACGCTCATCAAGGC | *HXT5* |
| 213 | Hx5ur | TTCGTACGCGTCTTCCGGAGTAACATGAAACCAGAGTACCACG | *HXT5* |
| 229 | Hx7df | AGAAGACCCTCGAGACGTCCGACGCTGAAGAAATGACTCACG | *HXT7* |
| 230 | Hx7dr | AGTCGACGGATCCGTAATTTTTCTTCTTTTAAGTGACGGGCG | *HXT7* |
| 243 | Gal2ufn | AAGCGGCCGCACTAGTACCGGTGATCTATATTCGAAAGGGGCGG | *GAL2* |
| 244 | Gal2urn | AACGTACGTCCGGATCATTAGAATACTTTTGAGATTGTGCGCT | *GAL2* |
| 233 | Ga2df | AGAAGACCCTCGAGACGTCTTACCTTGGAAATCTGAAGGCTGG | *GAL2* |
| 234 | Ga2dr | GTGGATCCTAGGTAAAACGGTACGAGAAAAGCTCCG | *GAL2* |

**Supplemental Table 3.** Oligonucleotides used for construction hexokinase deletion strain

| Number | Primer | Sequence (5’🡪 3’) | Gene(s) |
| --- | --- | --- | --- |
| 834 | Hxk2f | GCCAGAAAGGGTTCCATGGCCGATGTGCCAAAGGAATTGATGCAACAAATCCGTCGACCTCGAGTACCGTTCG | *HXK2* |
| 835 | Hxk2r | GCCAGAAAGGGTTCCATGGCCGATGTGCCAAAGGAATTGATGCAACAAATCCGTCGACCTCGAGTACCGTTCG | *HXK2* |
| 838 | Glk1f | ATGTCATTCGACGACTTACACAAAGCCACTGAGAGAGCGGTCATCCAGGCCCGTCGACCTCGAGTACCGTTCG | *GLK1* |
| 839 | Glk1r | CAATCTTCAAGTGCACCTTCCTCTCACCCTCGGCACCCAAGGGTGACAAGCCGGATCCTACCGTTCGTATAGC | *GLK1* |
| 846 | Hxk1f | ATGGTTCATTTAGGTCCAAAGAAACCACAGGCTAGAAAGGGTTCCATGGCCGGATCCACTAGCATAACTTCG | *HXK1* |
| 847 | Hxk1r | ATGGTTCATTTAGGTCCAAAGAAACCACAGGCTAGAAAGGGTTCCATGGCCGGATCCACTAGCATAACTTCG | *HXK1* |
| 848 | Gal1f | ATGACTAAATCTCATTCAGAAGAAGTGATTGTACCTGAGTTCAATTCTAGCGGATCCACTAGCATAACTTCG | *GAL1* |
| 849 | Gal1r | TTATAATTCATATAGACAGCTGCCCAATGCTGGTTTAGAGACGATGATAGTTGGGCCGCCAGTGTGATGG | *GAL1* |

**Supplemental Table 4.** Oligonucleotides used in qPCR.

| Name | Sequence (5’ 🡪 3’) |
| --- | --- |
| ActinF  ActinR  HXT1F  HXT1R  HXT2F  HXT2R  HXT3F  HXT3R  HXT4F  HXT4R  HXT5F  HXT5R  HXT7F  HXT7R  HXT8F  HXT8R  HXT9F  HXT9R  HXT10F  HXT10R  HXT11F  HXT11R  HXT12F  HXT12R  HXT13F  HXT13R  HXT14F  HXT14R  HXT15F  HXT15R  HXT16F  HXT16R  HXT17F  HXT17R  GAL2F  GAL2R | GGATTCTGAGGTTGCTGCTTTGG  GAGCTTCATCACCAACGTAGGAG  TGTTCTCTGTACACCGTTGACCG  AGATCATACAGTTACCAGCACCC  CTTCGCATCCACTTTCGTG  AATCATGACGTTACCGGCAGCC  GAAGCTAGAGCTGCTGGTTCAGC  ACAACGACATAAGGAATTGGAGCC  ATGGAGAGTTCCATTAGGTCTAGG  ATAACAGCTGGATCGTCTGCGC  TTGCTATGTCGTCTATGCCTCTG  AGATAAGGACATAGGCAACGGG  GGGTGCTGCATCCATGACTGC  ACAACGACATAAGGAATTGGAGCC  GTACTACTATCTTCAAATCTGTCGG  CTTGTGACGCCAACGGAGGCG  CCATTGAGAGGTTTGGACGCCG  ACACAATCATACAGTTACCGGCG  GGAATGCAAGACTCTTTCGAGAC  CTAGTGACGCCAACGGTGGCG  GCCACTCAATGGAGAGTCGGC  CAACTAGCAAGGCTGGATCGTC  CACCATCTTCAAATCTGTCGGTC  CAATCATACAGTTACCGGCACCC  CCCTCATGGCCAGGACGGTC  TTGCCATAACCAGTTGCATGCAG  GCCTTAGTAGTGTACTGCATCGGT  TGATACGTAGATACCATGGAGCC  GAGGCCTGTGTCTCCATCGCC  CACAAGAATACCTGTGATCAAACG  CAAGGAAGTATAGTAATACTGCGC  TTGGCGATGGAGACACAGGCC  TAACACTGCACAATGGAGAGTCC  TGAGTACCCATGGATCCTCTGG  TCAATGGAGAGTTCCATTAGGGC  CTGGACGGCAGGATCCTCTGG |

**Supplemental Table 5.** Oligonucleotides used in cloning and sequencing.

| Name | Sequence (5’ 🡪 3’) |
| --- | --- |
| F HXT1 Xbai | GCATTCTAGAATGAATTCAACTCCCGATCTAATATC |
| R HXT1 Cfr9i | TGCATCCCGGGTTATTTCCTGCTAAACAAACTCTTGTA |
| F HXT2 Xbai | GTCCTCTAGAATGTCTGAATTCGCTACTAGCCG |
| R HXT2 Cfr9i | CATCGCCCGGGTTATTCCTCGGAAACTCTTTTTTCTTTTG |
| F HXT36 Bcui | GCATACTAGTATGAATTCAACTCCAGATTTAATATCTC |
| R HXT36 BamHi | ACGTGGATCCTTATTTGGTGCTGAACATTCTCTTGT |
| R HXT36 BamHI-stop | CCATGGATCCTTTGGTGCTGAACATTCTCTTGTAC |
| F HXT4 Xbai | GTCCTCTAGAATGTCTGAAGAAGCTGCCTATCAAG |
| R HXT4 RN Cfr9i | TATCGCCCGGGTTAATTAACTGACCTACTTTTTTCCGA |
| F HXT5 Xbai | GTCCTCTAGAATGTCGGAACTTGAAAACGCTCATC |
| R HXT5 Cfr9i | GCATCCCGGGTTATTTTTCTTTAGTGAACATCCTTTTATA |
| F HXT7 Xbai | GTCCTCTAGAATGTCACAAGACGCTGCTATTGCA |
| R HXT7 Cfr9i | CATCGCCCGGGTTATTTGGTGCTGAACATTCTCTTG |
| F saci s promHXT7 | ATCGTCTAGATCTCGTAGGAACAATTTCGGGCCC |
| R promHXT7 xbai | AGTCTCTAGATTTTTGATTAAAATTAAAAAAACTTTTTGTTTTTG |
| F terHXT7 Bsu15i | GCATATCGATTTTGCGAACACTTTTATTAATTCATGATC |
| R terHXT7 Sali | GCATGTC GACGCAAGAACCATAATCCTCCTTTCTG |
| F HXT36 367NNN | CGGTGTCGTCnnnTTCTTCTCTACTTGTTG |
| R HXT36 367NNN | CAACAAGTAGAGAAGAAnnnGACGACACCG |
| F GFP BamHI | AAAGGATCCATGGTGAGCAAGGGCGAGGAGC |
| R GFP ClaI | AAAATCGATTTACTTGTACAGCTCGTCC |

n is any nucleotide

**Supplemental Table 6.**

**Hxt36 Hxt36-N367I Hxt36-N367A**

AVG STD AVG STD AVG STD

Residual sugar (g/l) 1.88 0.01 6.74 0.21 0.08 0.01

Q D-glucose ^a^ (g D-glc/gDW.h) 1.82 0.05 0.38 0.01 0.93 0.01

Q D-xylose ^b^ (g D-xyl/gDW.h) 0.15 0.01 0.12 0.01 0.27 0.01

Y EtOH (g EtOH/g sugar consumed) 0.39 0.01 0.43 0.01 0.41 0.01

Y EtOH (g EtOH/g total sugar) 0.32 0.01 0.17 0.01 0.40 0.01

^a^ D-glucose conversion rates were calculated in the first 1.5 hours

^b^ D-xylose conversion rates were calculated in the first 7.5 hours
